# Non-invasive preoperative prediction of surgical outcomes in drug-resistant epilepsy using seizure-related EEG network dynamics

**DOI:** 10.3389/fnins.2026.1941990

**Published:** 2026-09-18

**Authors:** Ruoxi Feng, Qiuchen Luo, Danni Yang, Weixin Kong, Yanming Han, Wentao Lin, Zhe Shi, Qiang Li

**Affiliations:** 1 School of Information Science and Engineering, Lanzhou University, Lanzhou, China; 2 School of Materials Science and Engineering, Lanzhou University of Technology, Lanzhou, China; 3 The Second Hospital & Clinical Medical School, Lanzhou University, Lanzhou, China; 4 The College of Biomedical Engineering, Fudan University, Shanghai, China

**Keywords:** brain networks, drug-resistant epilepsy, EEG source imaging, machine learning, surgical outcome prediction

## Abstract

Stereoelectroencephalography (SEEG)-guided radiofrequency thermocoagulation (RF-TC) has emerged as a minimally invasive treatment option for selected patients with drug-resistant epilepsy (DRE). However, its overall therapeutic efficacy remains suboptimal, with seizure-free rates typically falling below 70%. To facilitate precision management of DRE and reduce the potential risk of ineffective or high-risk surgical interventions, a reliable, robust, and non-invasive preoperative evaluation framework for accurately predicting postoperative surgical outcomes is critically needed. This study proposes a novel non-invasive computational framework based on EEG source imaging for preoperative outcome prediction by quantitatively characterizing seizure-related brain network reorganization. Scalp EEG recordings from 50 patients with DRE were reconstructed into source space and segmented into pre-ictal and ictal states. Functional brain networks were constructed based on clinically predefined epileptogenic zone (pEZ) and non-epileptogenic zone (pNEZ), preoperatively defined by clinical experts, and multidimensional seizure-related network features were then extracted for subsequent predictive modeling. The proposed framework achieved robust and stable performance across various network thresholds, with optimal results observed at intermediate sparsity levels. The best predictive performance was obtained using pEZ-derived features, achieving a maximum AUC of 0.936 and an accuracy of 0.860. Both support vector machine (SVM) and logistic regression (LR) models demonstrated competitive classification performance. pEZ-derived features achieved higher peak predictive performance, whereas pNEZ-derived features provided complementary information and demonstrated more stable performance across different thresholds. These findings collectively demonstrate that the proposed method enables accurate, reliable, and non-invasive preoperative prediction of surgical outcomes, thereby providing a practical decision-support tool for individualized surgical planning and effectively reducing the risk of potentially ineffective interventions.

## 1 Introduction

Epilepsy is a chronic neurological disorder characterized by recurrent, unprovoked seizures arising from hypersynchronous neuronal activity. Although pharmacological therapy remains the primary treatment, approximately one-third of patients develop drug-resistant epilepsy (DRE), for whom surgical resection or thermal ablation of the epileptogenic zone (EZ) offers the greatest likelihood of achieving seizure control (Bernasconi et al., 2019). However, postoperative seizure freedom is achieved only in a subset of patients, highlighting the need for reliable non-invasive biomarkers to improve preoperative evaluation (Figure 1).

**Figure 1:**
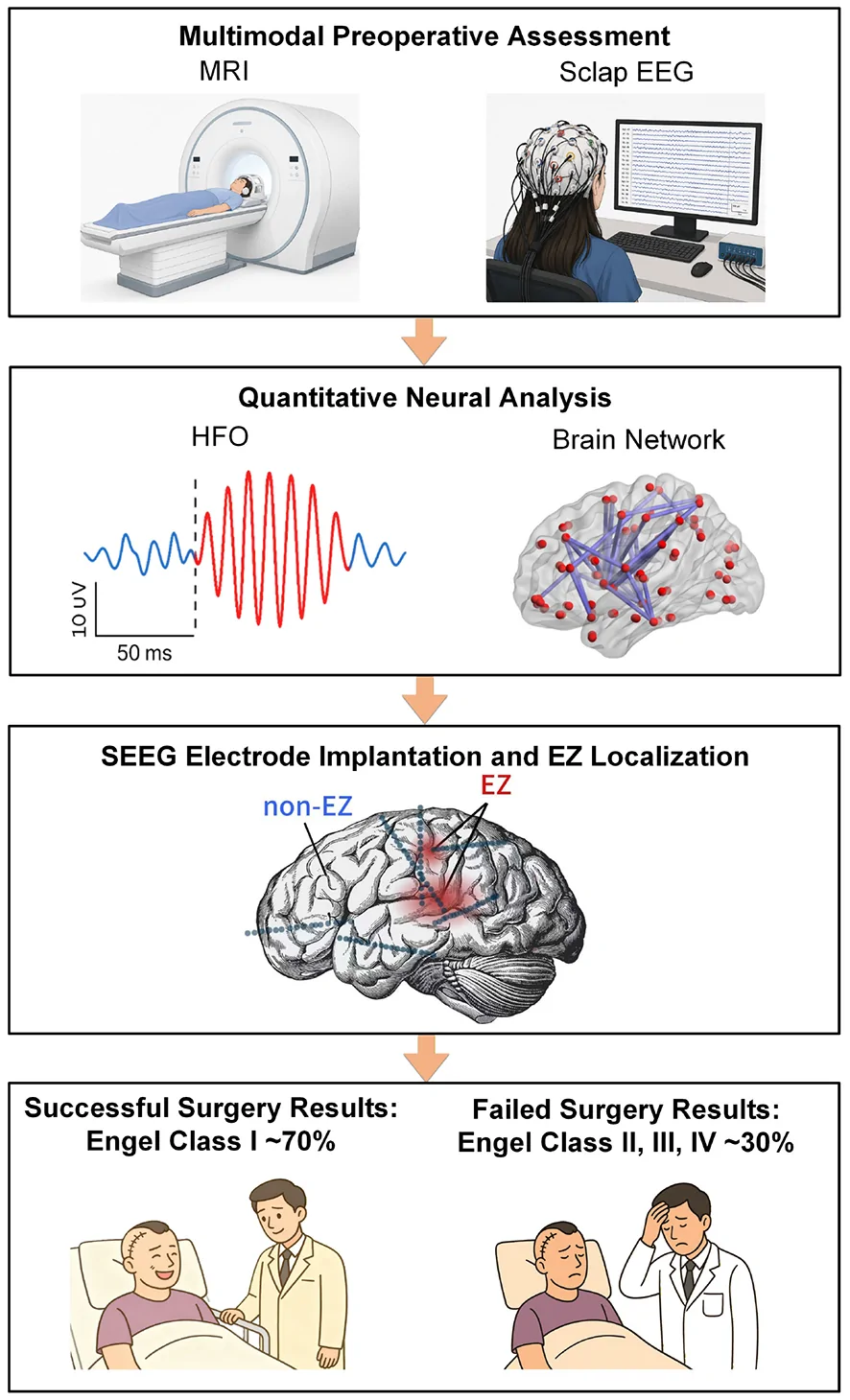
Clinical background of drug-resistant epilepsy (DRE), illustrating the typical clinical workflow from pre-operative evaluation to surgical treatment and postoperative outcomes.

Conventional preoperative assessment integrates semiology, structural imaging, and video-electroencephalography (vEEG). Although stereoelectroencephalography (SEEG) remains the clinical gold standard for EZ localization, its invasiveness and limited spatial sampling restrict widespread application. In contrast, source-level EEG reconstructed using standardized low-resolution electromagnetic tomography (sLORETA) offers a non-invasive approach to investigate whole-brain electrophysiological dynamics with high temporal resolution (Michel and Brunet, 2019; Wang et al., 2026). From a network neuroscience perspective, epilepsy is increasingly viewed as a disorder of large-scale brain networks, characterized by abnormal interactions among distributed regions (Abbott et al., 2019). Functional connectivity measures, such as the phase-lag index (PLI), are widely used in EEG-based network analysis due to their robustness against volume conduction effects (Stam et al., 2007).

Despite these advances, several limitations remain in current predictive frameworks. First, many studies focus on static connectivity patterns derived from either pre-ictal or ictal states, potentially overlooking dynamic network reorganization during seizure evolution (Cao et al., 2022; Lin et al., 2025). Second, the spatial heterogeneity of network alterations, particularly the distinct roles of the predefined epileptogenic zone (pEZ) and predefined non-epileptogenic zone (pNEZ), remains insufficiently characterized at the source level. Third, although machine learning techniques are increasingly applied, it remains unclear whether complex non-linear models consistently outperform simpler linear approaches for modeling seizure-related network features (Burns et al., 2014; Mercier et al., 2024; Wang et al., 2024b).

To address these issues, this study proposes a non-invasive preoperative framework for surgical outcome prediction based on seizure-related EEG network dynamics. Focusing on the transition between pre-ictal and ictal states, network reorganization is characterized using both difference (Δ) and relative ratio representations. In addition, spatially resolved analysis based on clinically pEZ and pNEZ regions is incorporated to capture region-specific contributions to outcome prediction. A systematic evaluation across multiple connectivity thresholds is performed to assess the robustness and generalizability of the proposed features. The overall goal is to provide an interpretable and effective network-based biomarker for clinical decision-making in patients with DRE (Figure 2).

**Figure 2:**
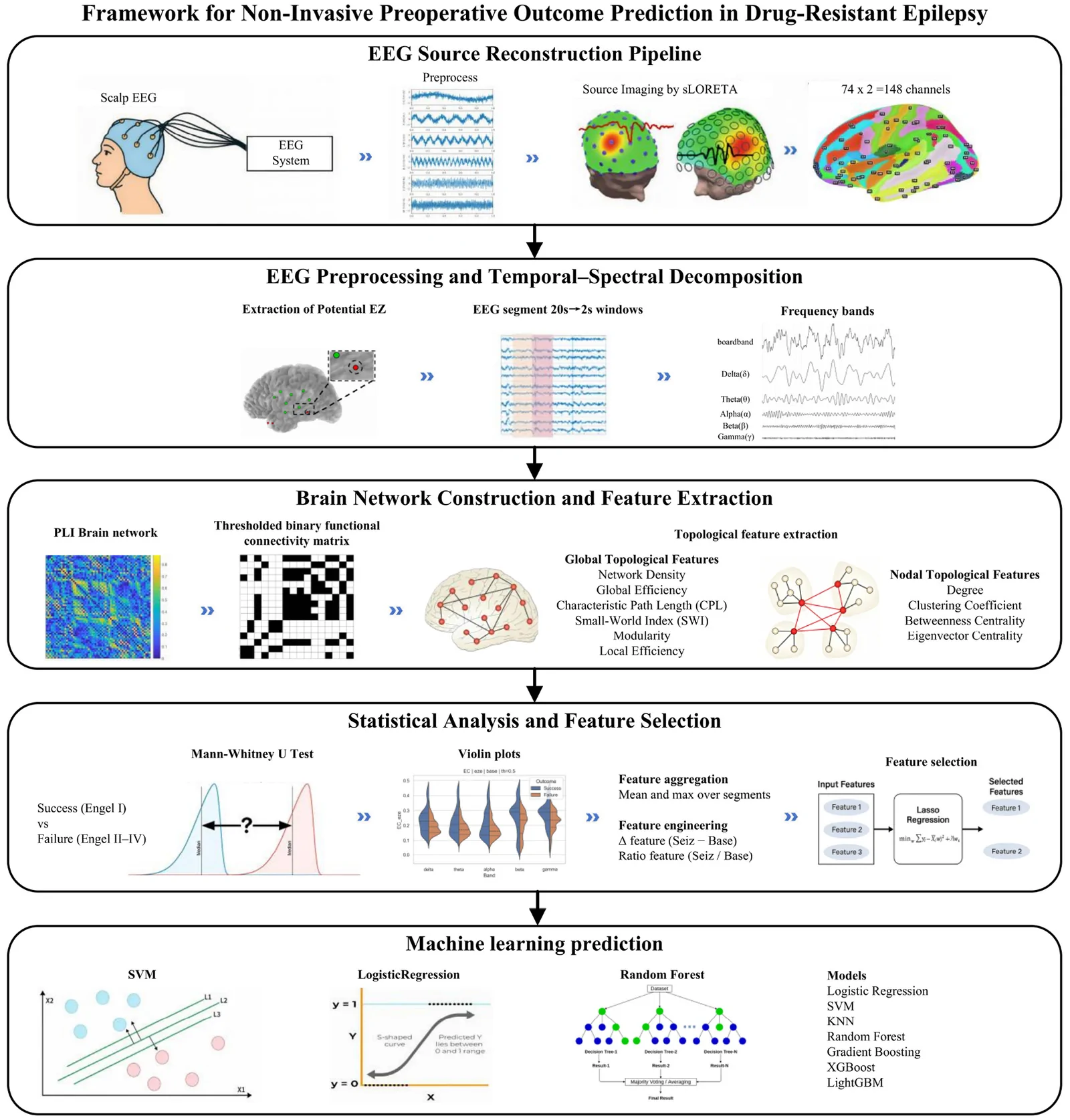
Overview of the proposed non-invasive framework for predicting surgical outcomes in drug-resistant epilepsy. The pipeline consists of EEG acquisition and preprocessing, source-level reconstruction, functional network construction using phase-lag index (PLI) with multi-thresholding, graph-theoretical feature extraction, statistical analysis, and machine learning-based classification.

## 2 Materials and methods

### 2.1 Patient population and clinical profiles

This retrospective study included 50 patients with drug-resistant focal epilepsy who underwent preoperative evaluation and subsequent surgical or ablative intervention (resection or SEEG-guided radiofrequency thermocoagulation) at the Second Hospital of Lanzhou University between 2022 and 2025 (Table 1). All clinical data were anonymized prior to analysis. The study was approved by the ethics committee of the Second Hospital of Lanzhou University (approval number: 2023A-765). All patients received standardized assessment, including structural MRI, neuropsychological evaluation, and long-term vEEG monitoring (sampling rate: 500 Hz). Clinical and demographic data, including age, sex, epilepsy duration, surgical modality, and lesion laterality, were collected. The pEZ was determined by integrating clinical semiology and electrophysiological findings (Table 1).

**Table 1:** Clinical and surgical characteristics of the patients.

ID	Gender	Age	Duration (years)	Surgical modality	Site	EEG	Engel
1	M	31	4	Resection	Right	2	I
2	F	12	7	Resection	Left	3	III
3	F	38	20	Thermocoagulation	Left	2	III
4	M	23	19	Resection+Thermocoagulation	Right	2	I
5	F	12	1	Thermocoagulation	Right	4	I
6	M	24	8	Thermocoagulation	Left	2	IV
7	F	28	16	Resection+Thermocoagulation	Left	3	I
8	M	36	18	Resection	Left	2	I
9	M	28	2	Resection	Left	4	II
10	M	23	7	Resection+Thermocoagulation	Right	4	I
11	M	15	12	Thermocoagulation	Right	5	I
12	M	17	5	Resection+Thermocoagulation	Left	6	I
13	M	18	3	Resection+Thermocoagulation	Right	4	III
14	M	30	5	Resection+Thermocoagulation	Left	2	I
15	M	24	12	Thermocoagulation	Left	2	IV
16	F	16	4	Thermocoagulation	Left	3	III
17	F	24	16	Thermocoagulation	Right	1	III
18	F	16	11	Thermocoagulation	Right	4	III
19	M	16	7	Thermocoagulation	Right	4	II
20	F	26	20	Thermocoagulation	Right	3	I
21	M	42	35	Thermocoagulation	Left	5	III
22	M	20	13	Resection	Left	5	I
23	M	22	15	Resection	Right	3	I
24	F	16	3	Resection	Right	2	I
25	F	36	21	Thermocoagulation	Left	3	III
26	M	12	5	Resection	Right	3	I
27	M	22	2	Thermocoagulation	Right	3	I
28	M	24	17	Resection+Thermocoagulation	Right	3	IV
29	M	19	13	Resection+Thermocoagulation	Left	4	IV
30	F	35	19	Thermocoagulation	Left	6	I
31	M	15	7	Resection	Left	3	II
32	M	27	25	Thermocoagulation	Left	2	III
33	M	28	24	Resection+Thermocoagulation	Left	3	I
34	F	31	26	Thermocoagulation	Left	4	I
35	F	32	21	Thermocoagulation	Left	3	II
36	M	29	15	Thermocoagulation	Right	2	III
37	F	17	15	Resection	Right	3	I
38	M	51	20	Thermocoagulation	Right	1	III
39	M	24	1	Thermocoagulation	Left	3	IV
40	M	36	–	Thermocoagulation	Right	4	IV
41	F	26	19	Thermocoagulation	Right	4	I
42	M	27	15	Thermocoagulation	Left	2	IV
43	M	37	2	Thermocoagulation	Right	3	I
44	F	20	2	Thermocoagulation	Right	3	I
45	M	18	8	Thermocoagulation	Right	2	I
46	M	26	20	Thermocoagulation	Right	3	IV
47	F	31	10	Resection+Thermocoagulation	Right	4	I
48	M	26	25	Resection	Right	5	I
49	M	22	13	Resection+Thermocoagulation	Left	4	I
50	M	13	10	Resection+Thermocoagulation	Right	1	II

M, male; F, female. Duration indicates the time from epilepsy onset to surgery. Thermocoagulation refers to SEEG-guided radiofrequency thermocoagulation. Engel classification: I, seizure free; II, significant reduction; III, slight reduction; IV, no improvement.

Postoperative seizure outcomes were assessed using the Engel classification system (Engel, 1993) after a mean follow-up duration of 2.84 years (range: 1–5 years), with a minimum follow-up of 12 months. For subsequent predictive modeling, patients were dichotomized into a surgical success group (Engel Class I, *n* = 26) and a surgical failure group (Engel Classes II–IV, *n* = 24). The mathematical definitions used throughout the analytical workflow are provided in Equations 1–16


$$y_{i}=\{\begin{array}{cc} 1, & \text{Engel Class I }\left(\text{seizure-free}\right)\\ 0, & \text{Engel Classes II–IV } \end{array}$$ (1)

where *y*_*i*_ denotes the surgical outcome of the *i*-th patient. This binary stratification is commonly adopted in machine learning-based outcome prediction studies (Sheikh et al., 2024).

To evaluate whether EEG-derived network features provide additional predictive value beyond conventional clinical information, a clinical baseline model was constructed using routinely available preoperative variables, including age, epilepsy duration, surgical modality, and lesion laterality.

### 2.2 EEG acquisition, source imaging, and preprocessing

Scalp EEG signals were recorded using a 148-channel EEG acquisition system with electrodes positioned according to the International 10–20 system. Raw recordings were visually inspected by experienced neurophysiologists to identify and exclude channels or segments contaminated by excessive muscle activity, motion artifacts, or amplifier saturation. The remaining signals were normalized using a *z*-score transformation to reduce inter-channel variability:


$$\begin{array}{c} z\left(t\right)=\frac{x\left(t\right)-\mu }{\sigma } \end{array}$$ (2)

where *x*(*t*) denotes the raw EEG signal, and μ and σ represent its mean and standard deviation, respectively.

EEG source imaging was performed using standardized low-resolution electromagnetic tomography (sLORETA), an MRI-constrained inverse solution method, to reconstruct cortical source activity (Dale and Sereno, 1993). Reconstructed signals were parcellated into anatomically defined regions of interest (ROIs), retaining only cortical gray matter sources. Each ROI was preoperatively classified by clinical experts as either pEZ or pNEZ based on multimodal evaluation, including electrophysiology, neuroimaging, and semiology (Shen et al., 2025).

To characterize state-dependent dynamics, all available seizures with reliable clinical annotations were included for each patient. The number of analyzed seizures varied across patients according to EEG recording availability. For patients with multiple seizures, network features were independently extracted from each seizure episode and subsequently averaged to obtain a single patient-level representation. For each seizure episode, two temporal epochs were defined: a 20-second pre-ictal epoch and a 20-second ictal epoch. The pre-ictal epoch was extracted from the interval between seizure onset and seizure occurrence, centered at the midpoint of the pre-seizure interval (i.e., onset/2 ± 10 s), whereas the ictal epoch was centered around the seizure midpoint (i.e., seizure midpoint ± 10 s). For each epoch, source-level time series were segmented into ten consecutive non-overlapping 2-second windows, denoted as ${x_{k}\left(t\right)}_{k=1}^{K}$, where *K* = 10 represents the number of segments. When multiple seizures were available for a patient, network features extracted from individual seizures were averaged to generate a single patient-level feature representation for subsequent machine learning analysis.

Each segment was decomposed into canonical frequency bands: delta (0.5–4 Hz), theta (4–8 Hz), alpha (8–13 Hz), beta (13–30 Hz), and gamma (30–80 Hz). Band-specific signals were obtained using zero-phase Butterworth bandpass filtering with forward–backward implementation to avoid phase distortion (Rousselet, 2012), preserving phase relationships for subsequent connectivity estimation.

The resulting frequency-resolved source signals served as input for functional connectivity analysis and network construction.

### 2.3 Analytical workflow and outcome definition

Frequency-specific functional connectivity was estimated at the source level using the PLI, which quantifies the asymmetry of phase differences between pairs of signals and reduces the influence of volume conduction (Stam et al., 2007). For each frequency band and 2-second temporal segment, PLI values were computed between all pairs of cortical regions to construct weighted adjacency matrices.

To ensure comparability across subjects and conditions, proportional thresholding was applied to the connectivity matrices. Specifically, a range of thresholds (τ = 0.3–0.8) was used to retain the strongest connections, resulting in sparse functional networks for subsequent analysis (van den Heuvel et al., 2017). Network construction was performed separately for the whole brain, pEZ, and pNEZ to capture spatially specific topological variations.

For each network, graph-theoretical metrics characterizing network integration and segregation were computed across frequency bands, temporal segments, and spatial domains.

To quantify seizure-induced network reorganization, difference and relative ratio features were defined between ictal and pre-ictal states:


$$\begin{array}{ccc} \Delta M & = & M_{\text{ictal}}-M_{\text{pre-ictal}}, \end{array}$$ (3)

$$\begin{array}{ccc} R_{M} & = & \frac{M_{\text{ictal}}}{M_{\text{pre-ictal}}+\epsilon }, \end{array}$$ (4)

where *M* denotes a network metric and ϵ is a small constant introduced to avoid numerical instability.

The resulting features were aggregated at the subject level across temporal segments and frequency bands to form a high-dimensional representation of network dynamics:


$$F_{i}=\left[\Delta M_{i},R_{i}\right],$$ (5)

where Δ**M**_*i*_ and **R**_*i*_ denote the concatenated difference and ratio features, respectively (Chen et al., 2025).

### 2.4 Network construction and feature extraction

#### 2.4.1 Phase-lag index

For each 2-second EEG source-level segment, the analytic phase ϕ_*i*_(*t*) of each cortical source signal was obtained using the Hilbert transform applied to zero-phase bandpass-filtered signals within canonical frequency bands (delta: 0.5–4 Hz, theta: 4–8 Hz, alpha: 8–13 Hz, beta: 13–30 Hz, and gamma: 30–80 Hz). All connectivity analyses were performed separately for each frequency band.

For any pair of cortical sources *i* and *j*, the instantaneous phase difference was defined as:


$$\begin{array}{c} \Delta \phi_{ij}\left(t\right)=\phi_{i}\left(t\right)-\phi_{j}\left(t\right). \end{array}$$ (6)

The phase-lag index (PLI) was calculated as:


$$\begin{array}{c} PLI_{ij}=|\frac{1}{T}\overset{T}{\underset{t=1}{\sum }}\text{sign}\left(\sin\left(\Delta \phi_{ij}\left(t\right)\right)\right)|, \end{array}$$ (7)

where *T* denotes the number of time samples.

To stabilize variance and improve numerical properties, PLI matrices were transformed using the Fisher *z* transformation:


$$\begin{array}{c} z_{ij}=\frac{1}{2}\ln\;\left(\frac{1+\text{PL}{\text{I}}_{ij}}{1-\text{PL}{\text{I}}_{ij}}\right). \end{array}$$ (8)

For each temporal segment and frequency band, symmetric connectivity matrices


$$\begin{array}{c} P\in {\mathbb{R}}^{N\times N} \end{array}$$ (9)

were constructed for the whole brain, as well as separately for the pEZ and pNEZ regions.

To control network sparsity and evaluate threshold robustness, connectivity matrices were thresholded using predefined network densities τ∈{0.3, 0.4, 0.5, 0.6, 0.7, 0.8} (Buchanan et al., 2020). For each threshold, binary adjacency matrices were generated as:


$$A_{ij}=\{\begin{array}{cc} 1, & z_{ij}\geq \tau \\ 0, & \text{otherwise} \end{array}$$ (10)

with self-connections removed (*A*_*ii*_ = 0). Proportional thresholding was applied to ensure comparable network density across subjects.

Network construction and subsequent graph-theoretical analyses were performed independently for each threshold, enabling systematic evaluation of the stability and robustness of network-derived features.

#### 2.4.2 Brain network metrics

Graph-theoretical metrics were calculated on undirected binary networks to characterize network integration, segregation, and centrality properties. Global measures included characteristic path length, global efficiency, network density, small-world index, and modularity, whereas local measures included node degree, clustering coefficient, betweenness centrality, eigenvector centrality, and local efficiency. These metrics are well-established measures in graph-theoretical network analysis, and their mathematical definitions are provided in the Supplementary material.

Node-level metrics were averaged within the whole brain, pEZ, and pNEZ regions to generate regional network features. Seizure-related difference and relative ratio features were subsequently derived from these network metrics as described in Section 2.3.

### 2.5 Machine learning classification

Postoperative outcome prediction was formulated as a binary classification task based on the Engel classification system, where Engel Class I was defined as a favorable surgical outcome and Engel Classes II–IV were considered unfavorable outcomes (Sheikh et al., 2024).

#### 2.5.1 Feature aggregation and representation

Segment-level network features were aggregated to construct subject-level representations. For each subject, frequency band, and seizure phase (pre-ictal and ictal), statistical summaries across all 2-second segments were calculated using both mean and maximum values:


$$\begin{array}{c} F^{\text{mean}}=\frac{1}{S}\overset{S}{\underset{s=1}{\sum }}F_{s},\;F^{\text{max}}=\underset{s}{\max}F_{s}, \end{array}$$ (11)

where *S* denotes the number of segments.

To characterize seizure-related network alterations, difference and relative ratio features were calculated as:


$$\begin{array}{c} \Delta F=F_{\text{ictal}}-F_{\text{pre}\text{-ictal}},\;R_{F}=\frac{F_{\text{ictal}}}{F_{\text{pre}\text{-ictal}}+\epsilon }, \end{array}$$ (12)

where ϵ represents a small constant to prevent numerical instability.

Features were calculated independently for each frequency band and concatenated into a subject-level feature vector:


$$\begin{array}{c} X_{i}=\left[\Delta F_{i}^{\delta },\ldots ,\Delta F_{i}^{\gamma },R_{F,i}^{\delta },\ldots ,R_{F,i}^{\gamma }\right]. \end{array}$$ (13)

This representation captures both absolute and relative alterations in functional network organization during seizure evolution.

#### 2.5.2 Feature preprocessing and selection

Before model training, missing values were imputed using the median value of each feature. Subsequently, all features were standardized to zero mean and unit variance:


$$\begin{array}{c} x_{j}^{\text{std}}=\frac{x_{j}-\mu_{j}}{\sigma_{j}}. \end{array}$$ (14)

Features with zero variance were removed to eliminate non-informative dimensions.

Feature selection was performed using L1-regularized logistic regression (LASSO) (Sun et al., 2024). This approach introduces sparsity by penalizing the absolute values of model coefficients:


$$\begin{array}{c} \hat{w}=\arg\underset{w}{\min}\left(L\left(w\right)+\lambda ||w|{|}_{1}\right). \end{array}$$ (15)

Only features with non-zero coefficients were retained, resulting in a compact and interpretable feature subset for subsequent classification.

#### 2.5.3 Classification and evaluation

Multiple supervised classifiers were evaluated, including linear models, distance-based algorithms, and tree-based ensemble methods, to capture both linear and nonlinear relationships within the extracted network features.

The evaluated classifiers included LR, linear support vector machine (SVM), K-nearest neighbors (KNN), random forest (RF), gradient boosting (GB), XGBoost, and LightGBM. Logistic regression was implemented with L2 regularization using the default solver (lbfgs) and class-balanced weighting. Linear SVM was implemented with a linear kernel and class-balanced weighting. KNN was configured using the default Euclidean distance metric. The RF classifier was configured with 500 trees, while GB used 300 estimators with a learning rate of 0.05. XGBoost was implemented with 300 estimators, a maximum tree depth of 4, a learning rate of 0.05, and sub-sampling ratios of 0.8 for both samples and features. LightGBM was configured with 300 estimators and 31 leaves. All experiments were implemented using Python 3.11 with the following scientific computing and machine learning packages: NumPy (v1.26.2), SciPy (v1.14.1), pandas (v2.2.3), scikit-learn (v1.5.2), NetworkX (v3.4.2), XGBoost (v3.1.1), and LightGBM (v4.6.0). Random seeds were fixed at 42 for all stochastic algorithms where applicable to ensure reproducibility.

No automated hyperparameter optimization was performed. Instead, predefined hyperparameters commonly used for biomedical classification tasks were adopted, and model comparison was conducted under the same repeated stratified 5-fold cross-validation framework.

Model performance was evaluated using repeated stratified 5-fold cross-validation with 10 repetitions, providing robust performance estimation while maintaining the original class distribution (Krstajic et al., 2014). All preprocessing procedures, including feature normalization and feature selection, were performed exclusively within the training folds to prevent information leakage.

Classification performance was assessed using accuracy, sensitivity, *F*_1_ score, and the area under the receiver operating characteristic curve (AUC):


$$\begin{array}{c} AUC=\overset{1}{\underset{0}{\int }}\text{TPR}\left(\text{FPR}\right)d\left(\text{FPR}\right). \end{array}$$ (16)

Final model performance was reported as the mean value across all cross-validation repetitions.

#### 2.5.4 Multi-threshold analysis

The complete machine learning pipeline was independently repeated for each network sparsity threshold (τ = 0.3–0.8). This multi-threshold strategy enabled systematic evaluation of model robustness across different network densities, which may substantially influence graph-theoretical properties and classification performance (Buchanan et al., 2020).

## 3 Results

### 3.1 Clinical baseline model

To quantify the additional predictive value of EEG network features, a clinical-only baseline model was evaluated using age, epilepsy duration, surgical modality, and lesion laterality. Using logistic regression with the same repeated stratified 5-fold cross-validation strategy, the clinical baseline achieved an AUC of 0.612 ± 0.169, an accuracy of 0.526 ± 0.143, a sensitivity of 0.503 ± 0.225, and an F1-score of 0.506 ± 0.184. These results indicate that conventional clinical characteristics alone provide limited predictive capability for postoperative outcome prediction, highlighting the potential added value of seizure-related EEG network representations. The performance of the clinical-only baseline model is summarized separately from EEG-based models to provide a reference for evaluating the incremental value of network-derived features.

### 3.2 Statistical analysis results

Non-parametric statistical analyses were performed to characterize differences in brain network organization between surgical outcome groups based on the Engel classification system, where Engel Class I indicates favorable outcome and Engel Classes II–IV represent unfavorable outcomes. Analyses were conducted across both pEZ and pNEZ regions, multiple proportional thresholds, and different frequency bands (Wang et al., 2024b).

Distributional characteristics of representative network features are illustrated in Figure 3. Across different thresholds (τ = 0.3–0.8), eigenvector centrality (EC) and modularity demonstrated consistent separation trends between outcome groups during the ictal phase (Guo et al., 2026). These differences were more evident in pEZ-derived features, suggesting that epileptogenic network alterations contain outcome-related information. In contrast, pNEZ-derived features showed more distributed patterns and may provide complementary information reflecting broader network reorganization. Their relatively homogeneous distributions across thresholds indicate stable network characteristics beyond the predefined epileptogenic regions (Shi et al., 2024).

**Figure 3:**
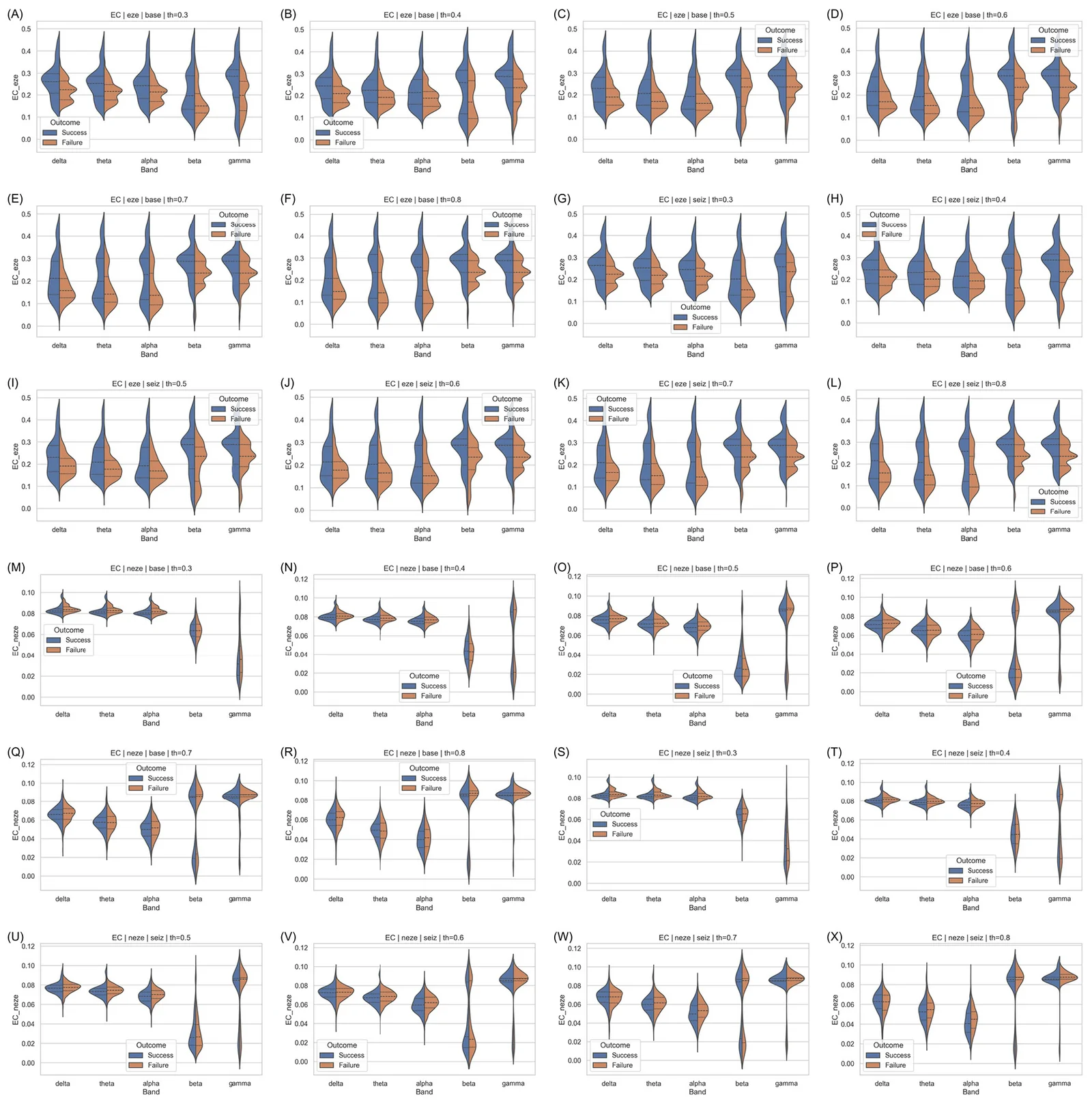
EC across different network sparsity thresholds (τ = 0.3–0.8), brain regions (pEZ and pNEZ), and temporal phases (pre-ictal and ictal). **(A–F)** pEZ during the pre-ictal phase at τ = 0.3–0.8; **(G–L)** pEZ during the ictal phase at τ = 0.3–0.8; **(M–R)** pNEZ during the pre-ictal phase at τ = 0.3–0.8; **(S–X)** pNEZ during the ictal phase at τ = 0.3–0.8. The distributions are compared between patients with favorable surgical outcomes (Engel Class I) and unfavorable outcomes (Engel Classes II–IV). The results demonstrate threshold-dependent and region-specific differences in network centrality patterns.

To further evaluate statistical consistency, *p*-value heatmaps across all features and frequency bands are presented in Figure 4. Significant group differences were observed across multiple frequency bands and thresholds in both regions. Notably, pNEZ features demonstrated more spatially widespread significance patterns (Pattnaik, 2025), whereas pEZ-related differences appeared more localized and threshold-dependent (Wang S. H. et al., 2024). These findings suggest that although seizure-related disruptions extend beyond the epileptogenic zone, outcome prediction may depend on both focal subnetworks and distributed network alterations rather than uniformly distributed changes across the brain.

**Figure 4:**
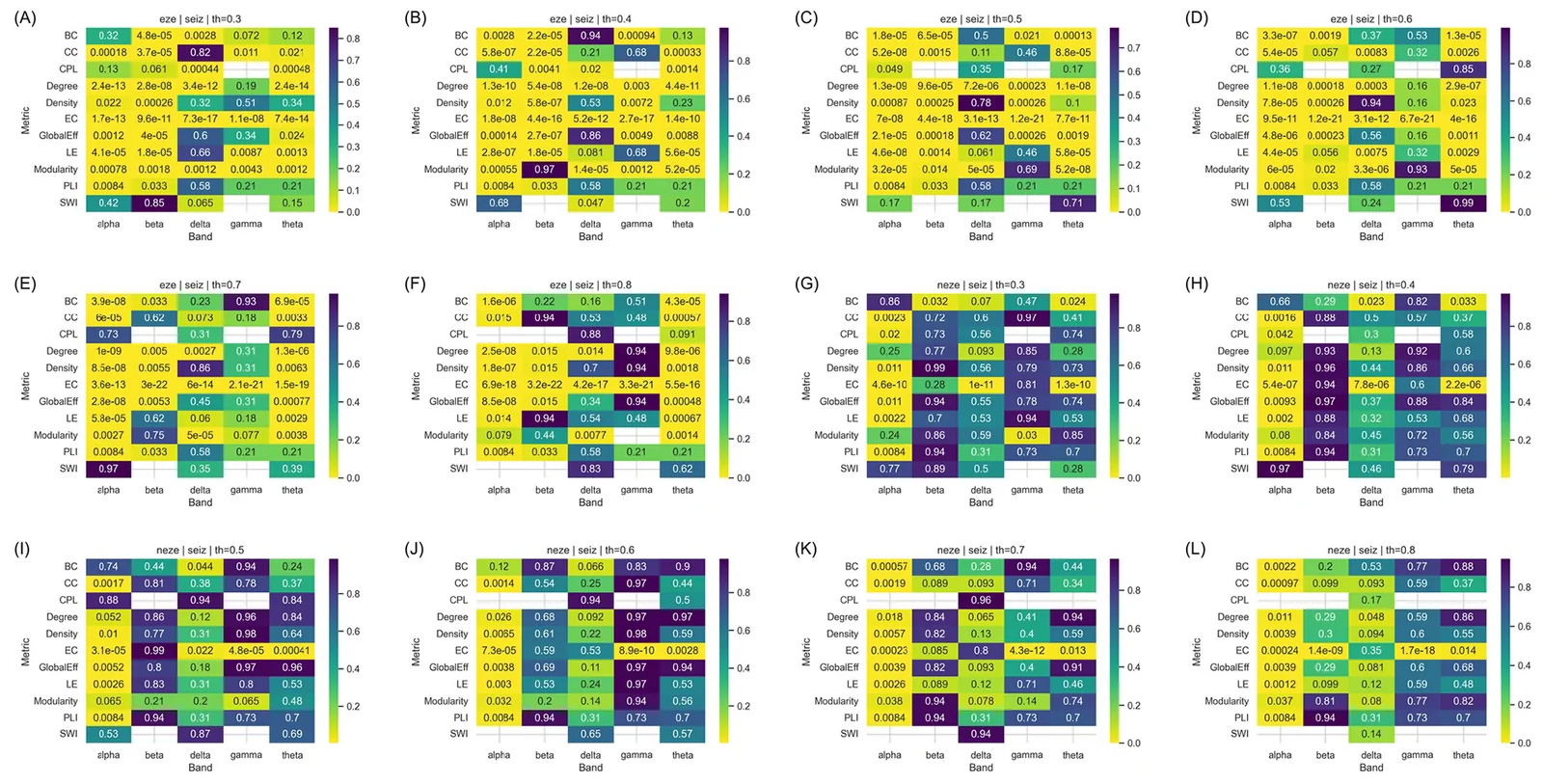
Heatmaps of statistical differences in network features across multiple sparsity thresholds (τ = 0.3–0.8). Results are presented separately for the pre-defined epileptogenic zone (pEZ) and pre-defined non-epileptogenic zone (pNEZ) during the ictal phase. **(A–F)** pEZ at τ = 0.3–0.8; **(G–L)** pNEZ at τ = 0.3–0.8. Color intensity represents the magnitude of statistical differences between favorable (Engel Class I) and unfavorable (Engel Classes II–IV) surgical outcome groups, highlighting spatial and threshold-dependent variations in discriminative network features.

Model-derived feature relevance is illustrated in Figure 5 using Random Forest feature importance distributions across different thresholds (Ntolkeras et al., 2024). Several network metrics, including EC, clustering coefficient (CC), and global efficiency, consistently exhibited high importance scores, consistent with previous graph-theoretical investigations of brain networks (Shi et al., 2024). Although pNEZ features showed relatively stable importance across thresholds, pEZ-derived features achieved higher peak importance values, particularly during the ictal phase. This suggests that focal network characteristics within epileptogenic regions may provide highly discriminative signals under optimized configurations, whereas distributed pNEZ features offer complementary information and contribute to robust prediction across different thresholds.

**Figure 5:**
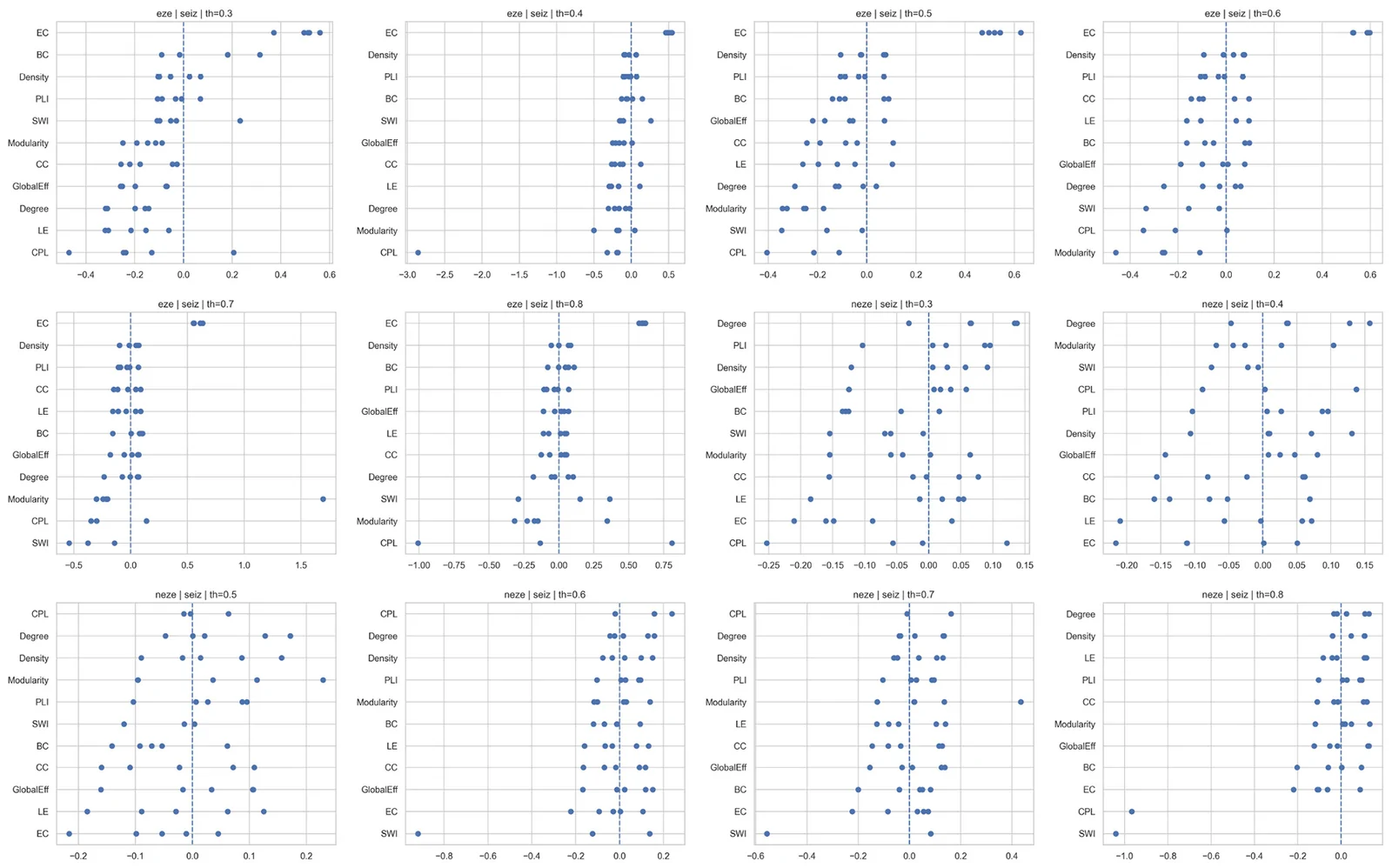
Feature importance analysis derived from Random Forest models across different network sparsity thresholds (τ = 0.3–0.8). Results are shown separately for pEZ and pNEZ during the ictal phase. Each panel represents the relative contribution of graph-theoretical network features to surgical outcome classification, identifying features with consistent predictive importance across different network configurations.

Across all thresholds, feature importance patterns and statistical differences remained largely consistent, indicating that the observed discriminative properties were not dependent on a specific sparsity level but reflected robust network characteristics. This threshold robustness agrees with previous graph-theoretical studies demonstrating relatively stable network properties across different network densities.

Frequency-specific analysis further revealed that high-frequency (γ band) features exhibited stronger and more consistent group differences compared with lower-frequency bands (Dallmer-Zerbe et al., 2025). In particular, γ-band EC and modularity demonstrated clearer separation trends and higher feature importance across thresholds. These findings support previous observations regarding the critical role of high-frequency activity in epileptogenic network dynamics and seizure propagation.

Overall, these results demonstrate that seizure-related network features capture both focal and distributed alterations in brain organization (Corona et al., 2023). While pNEZ regions exhibited widespread and relatively stable network alterations, pEZ-derived features showed higher discriminative potential under optimized network configurations. Together, these findings indicate that focal epileptogenic abnormalities and distributed network reorganization provide complementary information for surgical outcome prediction. This complementary pattern supports the interpretation of epilepsy as a large-scale network disorder, with surgical outcomes reflecting both focal epileptogenic abnormalities and distributed network reorganization.

### 3.3 Surgical outcome prediction results

The performance of machine learning models for postoperative surgical outcome prediction was systematically evaluated across proportional thresholds ranging from τ = 0.3 to 0.8, as summarized in Table 2. All models were trained using seizure-related features derived from graph-theoretical network metrics, where surgical success was defined as Engel Class I and surgical failure was defined as Engel Classes II–IV. Model evaluation was performed at the patient level using stratified cross-validation, and performance was quantified using AUC, accuracy, sensitivity, and F1-score (Tang et al., 2022).

**Table 2:** Best classification performance across different sparsity thresholds (τ = 0.3–0.8) using seizure-related features.

Threshold	Region	Model	AUC	Accuracy	Sensitivity	F1-score
0.3	ALL	SVM	0.849	0.806	0.838	0.811
0.3	pEZ	SVM	0.864	0.790	0.783	0.790
0.3	pNEZ	LR	0.778	0.678	0.648	0.662
0.4	ALL	SVM	0.868	0.892	0.831	0.882
0.4	pEZ	SVM	0.826	0.722	0.658	0.695
0.4	pNEZ	SVM	0.792	0.706	0.709	0.710
0.5	ALL	SVM	0.839	0.840	0.813	0.838
0.5	pEZ	LR	0.873	0.798	0.820	0.805
0.5	pNEZ	LR	0.719	0.682	0.657	0.674
0.6	ALL	LR	0.841	0.834	0.852	0.838
0.6	pEZ	LR	0.936	0.860	0.859	0.863
0.6	pNEZ	LR	0.739	0.664	0.701	0.677
0.7	ALL	LR	0.850	0.860	0.859	0.859
0.7	pEZ	LR	0.821	0.722	0.698	0.715
0.7	pNEZ	LR	0.805	0.738	0.768	0.746
0.8	ALL	LR	0.887	0.842	0.899	0.862
0.8	pEZ	LR	0.759	0.690	0.648	0.679
0.8	pNEZ	SVM	0.806	0.708	0.686	0.696

For each threshold and region (ALL, pEZ, and pNEZ), the best-performing model is reported.

At lower thresholds, strong and consistent discriminative performance was observed. At τ = 0.3, SVM achieved the best performance in the pEZ network (AUC = 0.864, accuracy = 0.790), whereas LR achieved the best performance in the pNEZ network (AUC = 0.778). At τ = 0.6, LR achieved the highest overall performance in the pEZ network (AUC = 0.936, accuracy = 0.860, F1-score = 0.862), representing the optimal classification performance across all evaluated thresholds. These findings indicate that seizure-related network alterations exhibit stable and approximately linearly separable characteristics under moderately sparse network configurations.

At intermediate thresholds (τ = 0.4 and 0.5), model performance remained robust with moderate variability across regions and classifiers. At τ = 0.4, SVM achieved the best performance in both pEZ (AUC = 0.826) and pNEZ (AUC = 0.792) networks, whereas at τ = 0.5, LR achieved the highest AUC in the pEZ network (0.873). These findings suggest that intermediate sparsity levels provide an optimal balance between network density and information preservation, supporting stable classification performance.

At higher thresholds, performance showed increased variability and region-specific differences. At τ = 0.7, LR achieved the best performance in the pNEZ network (AUC = 0.805), whereas pEZ performance decreased slightly (AUC = 0.821). At τ = 0.8, SVM maintained competitive performance in the pNEZ network (AUC = 0.806), although overall performance declined compared with moderate thresholds (Mercier et al., 2025). This non-monotonic trend suggests that extremely dense or sparse network representations may reduce feature stability, whereas appropriate sparsity levels enhance discriminative signals by suppressing weak connections.

Across all thresholds, several consistent patterns were identified. First, LR demonstrated stable and frequently optimal performance, particularly for pEZ-derived features, which achieved the highest peak predictive performance at τ = 0.6 (AUC = 0.936). However, this peak performance was threshold-dependent, whereas pNEZ-derived features showed relatively stable predictive performance across different sparsity levels. Second, SVM showed robust performance across different sparsity conditions (Mercier et al., 2025). In contrast, ensemble models, including Random Forest and XGBoost, did not consistently outperform linear classifiers, suggesting that the extracted network features form a relatively structured and low-dimensional representation.

Feature selection analysis further demonstrated stable patterns across thresholds. LASSO identified a compact subset of discriminative features at each sparsity level. Features derived from both pEZ and pNEZ regions were consistently retained, including clustering coefficient, modularity, characteristic path length, and eigenvector centrality, reflecting complementary aspects of network segregation and integration. The persistence of these features across thresholds indicates their robust association with postoperative surgical outcomes.

Overall, seizure-related EEG network features provided reliable predictive information for surgical outcome classification. The threshold-dependent performance highlights the importance of appropriate network construction, while the strong performance of linear classifiers suggests a stable and interpretable feature space. These findings support non-invasive EEG-based network analysis combined with machine learning as a potential framework for epilepsy surgery outcome prediction.

#### 3.3.1 Feature-group ablation analysis

To further investigate the contribution of temporal representations and spatial feature sources, an ablation analysis was performed using the same machine learning pipeline and cross-validation strategy.

For temporal representation analysis, static pre-ictal and ictal features were compared with seizure-related dynamic representations, including ictal-preictal difference (Δ), ratio, and combined Δ+ratio features. All temporal ablation experiments were performed using whole-brain (ALL) features to ensure a fair comparison (Table 3).

**Table 3:** Feature-group ablation analysis for surgical outcome prediction.

Category	Feature group/source	Representation	Region	Best classifier	AUC/accuracy
Panel A: Temporal representation					
Temporal	Pre-ictal	Static	ALL	LR	0.813/0.728
Temporal	Ictal	Static	ALL	LR	0.800/0.702
Temporal	Δ	Dynamic	ALL	SVM	0.802/0.738
Temporal	Ratio	Dynamic	ALL	SVM	0.871/0.806
Temporal	Δ+Ratio	Dynamic	ALL	LR	0.887/0.842
Panel B: Spatial contribution					
Spatial	ALL	Δ+Ratio	Whole brain	LR	0.887/0.842
Spatial	pEZ	Δ+Ratio	pEZ	LR	0.773/0.692
Spatial	pNEZ	Δ+Ratio	pNEZ	SVM	0.806/0.708

Temporal representation ablation compares static pre-ictal and ictal features with seizure-related dynamic features. Spatial contribution analysis evaluates the predictive value of complete, pEZ-derived, and pNEZ-derived feature sets. All experiments used the same machine learning pipeline and cross-validation strategy.

As shown in Table 3, static pre-ictal and ictal representations achieved AUC values of 0.813 and 0.800, respectively. Dynamic representations provided improved predictive performance, particularly the ratio and combined Δ+ratio features, which achieved AUC values of 0.871 and 0.887. These results indicate that characterizing seizure-related network transitions provides additional predictive information beyond individual temporal states.

For spatial contribution analysis, the optimal temporal representation (Δ+Ratio) was further evaluated using different spatial feature sources. The complete feature set (ALL) achieved the highest performance (AUC = 0.887), while pNEZ-derived features achieved competitive performance (AUC = 0.806) and pEZ-derived features achieved an AUC of 0.773 under this ablation setting.

Although pEZ-derived features showed lower performance in this controlled ablation comparison, they achieved higher peak predictive performance under optimized network threshold configurations in the multi-threshold analysis (Table 2). In contrast, pNEZ-derived features demonstrated more stable performance across different thresholds, suggesting complementary predictive information from distributed network alterations.

## 4 Discussion

This study presents a non-invasive EEG source imaging framework for predicting postoperative outcomes in drug-resistant epilepsy by characterizing seizure-related network reorganization (Mercier et al., 2025). By integrating frequency-specific functional connectivity analysis, multi-threshold graph-theoretical characterization, and machine learning models, the proposed framework extracted clinically relevant predictive features from both ictal and pre-ictal states. The results demonstrate that surgical outcomes are associated with distributed network alterations rather than focal epileptogenic activity alone, supporting a network-based understanding of epilepsy. The ablation analysis further demonstrated that dynamic seizure-related representations provided greater predictive value than static pre-ictal or ictal network features. Moreover, the complementary contributions of pEZ and pNEZ features suggest that outcome prediction depends on both focal epileptogenic alterations and distributed network reorganization.

### 4.1 Distributed network reorganization beyond the epileptogenic zone

pNEZ-derived features demonstrated relatively stable predictive performance across different network thresholds, suggesting that distributed network alterations contain robust outcome-related information. In contrast, pEZ-derived features, although more sensitive to threshold selection, achieved higher peak predictive performance under specific network configurations. Favorable outcomes (Engel Class I) were associated with relatively more constrained network reorganization, whereas unfavorable outcomes (Engel Classes II–IV) showed broader alterations involving distributed epileptic networks. These observations suggest that surgical failure may result from incomplete modification of pathological networks beyond the primary epileptogenic region. The consistency across frequency bands and network densities further emphasizes the importance of integrating whole-brain network information.

### 4.2 Comparison with previous EEG/SEEG-based outcome prediction studies

Previous studies have investigated epilepsy surgery outcome prediction using both invasive SEEG recordings and non-invasive EEG signals. SEEG-based approaches benefit from high spatial resolution and direct sampling of epileptogenic regions, and several studies have demonstrated that interictal or ictal network properties can provide predictive information regarding postoperative seizure outcomes (Burns et al., 2014; Mercier et al., 2024; Wang et al., 2024b). However, the invasive nature of SEEG limits its application as a routine preoperative evaluation tool.

In contrast, EEG-based approaches provide a safer and more accessible alternative, but conventional scalp EEG studies have mainly focused on static biomarkers, including spectral features, connectivity strength, or individual network characteristics (Stam et al., 2007; Cao et al., 2022; Lin et al., 2025). These approaches may not fully capture the dynamic reorganization of epileptic networks during seizure evolution.

Compared with previous studies, the present work introduces a non-invasive source-space EEG network framework that characterizes seizure-related transitions between pre-ictal and ictal states (Lin et al., 2025; Wang et al., 2024a). Instead of relying on static connectivity patterns alone, we extracted dynamic network alterations using difference and relative ratio features across multiple sparsity thresholds. Furthermore, the analysis separately evaluated clinically predefined epileptogenic and non-epileptogenic regions, enabling investigation of both focal pathological activity and distributed network reorganization.

The proposed framework achieved a maximum AUC of 0.936 using pEZ-derived network features, showing comparable predictive performance to previous EEG-based outcome prediction approaches while avoiding the requirement for invasive electrode implantation. Importantly, the findings suggest that outcome prediction is not solely determined by local epileptogenic abnormalities, but also reflects broader alterations in large-scale brain network organization. Therefore, seizure-related network dynamics may provide complementary information for individualized surgical outcome assessment beyond conventional clinical and electrophysiological markers.

### 4.3 Frequency-specific dynamics and high-frequency activity

High-frequency (γ-band) activity demonstrated a prominent role in distinguishing surgical outcomes. Across analyses, γ-band network features, particularly eigenvector centrality and modularity, showed prominent discriminative patterns compared with lower-frequency bands (Raghavan et al., 2024). Increased high-frequency centrality in unfavorable outcomes may reflect enhanced synchronization and abnormal information propagation within epileptic networks, whereas favorable outcomes may correspond to more regulated network configurations. Seizure-related features derived from both ictal and pre-ictal states were essential for capturing these dynamic alterations.

### 4.4 Threshold stability and network representation

Multi-threshold analysis demonstrated that predictive performance was not monotonically associated with network sparsity. The optimal performance was achieved at an intermediate threshold (τ = 0.6 for pEZ), suggesting a balance between retaining meaningful network connections and reducing noise. Although classification performance varied across thresholds, statistical differences and feature importance patterns remained relatively consistent, particularly for pNEZ-derived features, indicating stable underlying network characteristics.

### 4.5 Linear separability and model interpretability

Linear models, particularly LR, consistently achieved the best or near-best performance across thresholds, reaching a peak AUC of 0.936 at τ = 0.6. More complex ensemble models did not provide consistent improvements, suggesting that seizure-related network features form a structured and separable representation. From a clinical perspective, linear models provide advantages in interpretability, stability, and transparency, facilitating potential integration into clinical decision-support systems.

### 4.6 Clinical implications

The proposed framework demonstrates that non-invasive EEG source imaging can capture clinically meaningful network biomarkers for predicting epilepsy surgery outcomes. Compared with invasive approaches, this method provides whole-brain electrophysiological characterization without additional surgical risk. Successful outcomes appear to depend not only on accurate epileptogenic zone identification but also on effective modulation of distributed pathological networks, providing new insights for individualized surgical planning. Compared with the clinical-only baseline model, which achieved limited predictive performance, EEG-derived network features provided substantial additional discriminative information. This finding suggests that seizure-related network dynamics capture complementary physiological information beyond conventional clinical characteristics, including age, epilepsy duration, surgical modality, and lesion laterality.

### 4.7 Limitations and future directions

Several limitations should be acknowledged. First, the sample size was moderate, which may limit the generalizability of the findings. Second, EEG source imaging is affected by spatial uncertainty arising from the ill-posed inverse problem. Third, although multi-threshold analysis demonstrated robustness, the optimal threshold selection strategy requires further validation. Future studies should include larger independent cohorts, integration of multimodal neuroimaging data, dynamic network analysis, and prospective clinical validation to evaluate the practical utility of the proposed framework.

## 5 Conclusion

This study demonstrates that seizure-related source-space EEG network dynamics provide a robust and non-invasive approach for preoperative prediction of surgical outcomes in drug-resistant epilepsy, with complementary contributions from focal epileptogenic and distributed network alterations supporting their potential utility in individualized surgical decision-making.

## Data Availability

The raw data supporting the conclusions of this article will be made available by the authors, without undue reservation.
