# Supplementary material for: Non-invasive preoperative prediction of surgical outcomes in drug-resistant epilepsy using seizure-related EEG network dynamics
Source: Front Neurosci. 2026 Sep 18;20:1941990. doi: 10.3389/fnins.2026.1941990 (PMC13630784; doi:10.3389/fnins.2026.1941990)
Supplement: Supplementary file 1 [file Data_Sheet_1.pdf]

SUPPLEMENTARY MATERIAL

SUPPLEMENTARY METHODS

S1. Definitions of Graph-Theoretical Network Metrics

Graph-theoretical metrics were calculated on undirected binary networks to characterize network integration, segregation, and centrality properties. The mathematical definitions of the global and local network metrics used in the main analysis are provided below.

S1.1 Global Network Measures

Characteristic Path Length

The characteristic path length was defined as:

$$L = \frac{1}{N(N-1)} \sum_{i \neq j} d_{ij}, \qquad \text{(S1)}$$

where N denotes the number of nodes and d_{ij} represents the shortest path length between nodes i and j.

Global Efficiency

Global efficiency was calculated as:

$$E_{\text{glob}} = \frac{1}{N(N-1)} \sum_{i \neq j} \frac{1}{d_{ij}}. \qquad \text{(S2)}$$

Network Density

Network density was defined as:

$$D = \frac{2E}{N(N-1)}, \qquad \text{(S3)}$$

where E denotes the number of edges.

Small-World Index

The small-world index was calculated using matched random networks with identical numbers of nodes and edges:

$$\text{SWI} = \frac{C_{\text{real}}/C_{\text{rand}}}{L_{\text{real}}/L_{\text{rand}}}, \qquad \text{(S4)}$$

where C_{real} and L_{real} denote the clustering coefficient and characteristic path length of the empirical network, respectively, and C_{rand} and L_{rand} denote the corresponding values of matched random networks.

Modularity

Modularity was calculated as:

$$Q = \frac{1}{2m} \sum_{i,j} \left(A_{ij} - \frac{k_i k_j}{2m} \right) \delta(c_i, c_j), \qquad \text{(S5)}$$

where m represents the total number of edges, A_{ij} denotes the element of the binary adjacency matrix, k_i and k_j denote node degrees, and $\delta(c_i, c_j)$ is the Kronecker delta function.

S1.2 Local Network Measures

Node Degree

Node degree was defined as:

$$k_i = \sum_j A_{ij}, \qquad \text{(S6)}$$

where A_{ij} denotes the element of the binary adjacency matrix.

Clustering Coefficient

The clustering coefficient was calculated as:

$$C_i = \frac{1}{k_i(k_i-1)} \sum_{j,k} (A_{ij} A_{ik} A_{jk})^{1/3}, \qquad \text{(S7)}$$

where k_i denotes the degree of node i.

Betweenness Centrality

Betweenness centrality was defined as:

$$BC_i = \sum_{s \neq i \neq t} \frac{\sigma_{st}(i)}{\sigma_{st}}, \qquad \text{(S8)}$$

where σ_{st} represents the number of shortest paths between nodes s and t, and $\sigma_{st}(i)$ denotes the number of those paths passing through node i.

Eigenvector Centrality

Eigenvector centrality was obtained by solving:

$$\mathbf{A}\mathbf{x} = \lambda \mathbf{x}, \qquad \text{(S9)}$$

where $\mathbf{A}$ denotes the adjacency matrix, $\mathbf{x}$ is the eigenvector centrality vector, and λ is the corresponding eigenvalue.

Local Efficiency

Local efficiency was defined as:

$$LE_i = \frac{1}{k_i(k_i - 1)} \sum_{j \neq k \in N(i)} \frac{1}{d_{jk}}, \tag{S10}$$

where $N(i)$ denotes the set of neighboring nodes of node i, and d_{jk} represents the shortest path length between nodes j and k within the subgraph induced by the neighbors of node i.

S1.3 Regional Feature Aggregation

Node-level metrics were averaged within the whole brain, pEZ, and pNEZ regions to generate regional network features.
